# Breaking the Stigma: A Systematic Review of Antipsychotic Efficacy in Children and Adolescents with Behavioral Disorders

**DOI:** 10.3390/medicines12030015

**Published:** 2025-06-23

**Authors:** Nuno Sanfins, Pedro Andrade, Jacinto Azevedo

**Affiliations:** 1 Faculty of Medicine, University of Porto, 4200-319 Porto, Portugal; pedrooliveiraandrade2001@gmail.com; 2 Department of Clinical Neuroscience and Mental Health, 4480-565 Touguinhó, Portugal

**Keywords:** oppositional defiant disorder, conduct disorder, antipsychotic treatment, atypical antipsychotics, behavioral disorders, disruptive behavior, systematic review

## Abstract

**Background/Objectives**: Oppositional defiant disorder (ODD) and conduct disorder (CD) are important behavior disorders in children and adolescents, often linked with long-term psychosocial problems. Antipsychotics are frequently prescribed to manage severe symptoms and improve behavior, but their efficacy in this population is still unclear and a lot of physicians are remittent in prescribing them. This systematic review aims to assess the effectiveness of antipsychotic treatment in reducing symptoms associated with ODD and CD in children and adolescents. **Methods**: Studies that investigated how effective antipsychotic treatments are for children and teens diagnosed with oppositional defiant disorder (ODD) and conduct disorder (CD) were reviewed. Only studies that met a few main criteria were included: participants were between 5 and 18 years old with an ODD or CD diagnosis; the treatment could be any type of antipsychotic, whether typical or atypical; the accepted study designs were randomized controlled trials (RCTs), cohort studies, systematic reviews with meta-analysis, or observational studies. The outcomes of interest were reductions in aggressive or defiant behaviors, improvements in social functioning, and the occurrence of any adverse effects from the medications. There was no restriction on the language of publication, and studies published from 2000 to 2024 were considered. Studies that focused only on non-antipsychotic drugs or behavioral therapies, as well as case reports, expert opinions, and non-peer-reviewed articles did not meet the inclusion criteria. **Results**: The review consisted of 13 studies. The results suggest that some antipsychotic drugs—especially atypical antipsychotics—can substantially reduce aggressive and defiant behavior in children and adolescents who have oppositional defiant disorder (ODD) or conduct disorder (CD). Common side effects of these medications include weight gain, sedation, and metabolic problems. **Conclusions**: Although adverse effects are a concern, the potential of these medications to manage disruptive behaviors should not be overlooked. When used in combination with behavioral therapy and other forms of treatment, antipsychotics can markedly improve the outcomes of these very difficult-to-treat patients. Clinicians who treat these patients need to consider antipsychotics as a serious option. If they do not, they are denying their patients medication that could greatly benefit them.

## 1. Introduction

Conduct disorder (CD) and oppositional defiant disorder (ODD) are disruptive behavior disorders that occur in children and adolescents. They are among the most common mental disorders affecting young people [1]. These two disorders appear to be closely related, and children and adolescents diagnosed with them frequently exhibit aggressive, defiant, and antisocial behaviors [2]. These behaviors can lead to many significant challenges in a young person’s life, including academic problems, trouble with family and friends, and an increased risk of serious antisocial behavior and criminal activity in the future [3]. The treatment of these disorders is multifaceted, with behavioral therapies, family interventions, and medications being cornerstone approaches [4]. Among pharmacological treatments, antipsychotic medications have been widely used, particularly for individuals with more severe presentations of aggression and defiance [5].

The debate is ongoing over how well disruptive behaviors of children with oppositional defiant disorder (ODD) and conduct disorder (CD) respond to treatment with antipsychotic medications [6]. Atypical antipsychotic drugs (e.g., risperidone, aripiprazole), which have been more recently developed, do show some promise in reducing aggression and improving overall behavior, at least in the short term [7]. But what does the use of these drugs in children portend for both mental and physical health? Concerns have been raised about potential side effects including (but not limited to) weight gain, sedation, and disturbances of metabolism [8].

Given the rather tenuous evidence and the potential dangers linked to antipsychotic treatment, it is important to determine whether these medications are effective for something like oppositional defiant disorder (ODD) or conduct disorder (CD). The purpose of this systematic review is to determine if antipsychotic drugs do (or do not) reduce disruptive behaviors. A secondary goal is to assess their corresponding side effects.

## 2. Materials and Methods

This systematic review was conducted following the PRISMA (Preferred Reporting Items for Systematic Reviews and Meta-Analyses) guidelines to ensure transparency, rigor, and methodological quality. The review protocol was registered with PROSPERO (ID: CRD420251009839).

The review encompassed studies that assessed the effectiveness of antipsychotic medications when given to children and adolescents with ODD and CD. Eligible studies had to meet a set of criteria:

1. Population: The studies had to involve participants who were children or adolescents (aged 5 to 18) with a diagnosis of ODD or CD.
2. Intervention: The studies had to evaluate the administration of any type of antipsychotic medication, whether typical or atypical.
3. Study Design: Only randomized controlled trials (RCTs), cohort studies, systematic reviews with meta-analysis, and observational studies were included.
4. Outcome Measures: The studies had to report some measures that indicated how well the medication was working.
5. Publication Language: The studies could be published in any language. If any study was published in a language in which the authors were not fluent, translation tools (like DeepL) were used.
6. Date: The studies could have any date of publication from 2000 to 2024.

Studies were excluded based on the following criteria:

1. Population: Studies where ODD and/or CD were not the primary diagnosis. Studies where individuals had low IQ (<70) or comorbid autism spectrum disorder (ASD).
2. Intervention: Studies that focused solely on non-antipsychotic pharmacological interventions or solely on behavioral therapies.

A thorough literature search using an electronic database search was performed to find studies that assess the effectiveness of antipsychotic drugs in treating youths diagnosed with ODD and CD. The subsequent electronic databases were investigated:

- PubMed/MEDLINE
- PsycINFO
- Cochrane Library
- EMBASE
- Web of Science

The terms of the search combined keywords and medical subject headings (MeSH) that pertained to the population, interventions, and outcomes of interest. The precise search string that was used was

(“Oppositional Defiant Disorder” OR “Conduct Disorder”)

AND

(“antipsychotic treatment” OR “atypical antipsychotics” OR “Risperidone” OR “Aripiprazole”)

AND

(“children” OR “adolescents”)

AND

(“aggression reduction” OR “behavioral improvement”)

To find more studies that possibly fell outside the database searches, a tool called Connected Papers was used. Connected Papers is a visual tool that maps the relationships between scientific papers based on co-citation and bibliographic coupling. This tool helped uncover related studies by visualizing networks of research papers connected to the key articles already identified. The following steps were taken using Connected Papers:

- Seminal papers identified from the initial search were input into the tool.
- The network of connected papers was explored to find additional relevant studies.
- The titles and abstracts of these connected papers were screened for inclusion.

The reference lists of all included studies and pertinent systematic reviews were manually searched to find additional studies that met our inclusion criteria.

All identified studies underwent a two-step screening process. To start, the titles and abstracts of all articles were reviewed to determine relevance to the inclusion and exclusion criteria. Articles that passed this first test were then reviewed in full—two reviewers working independently decided on each article’s eligibility. Where the reviewers’ judgments did not agree, the two first discussed the case. If they could not come to a consensus, a third reviewer was brought in to break the tie.

A standardized form was used to extract the data. The data extracted included

- The study characteristics (author, year, country, study design).
- The characteristics of the participants (age, gender, diagnostic criteria, sample size).
- The intervention details (type of antipsychotic, dosage, treatment duration).
- The outcome measures (changes in behavior, social functioning, adverse effects).
- The results (statistical findings, effect sizes, significance levels).

Two reviewers performed the data extraction independently. Discrepancies were resolved by consensus.

In this review, the risk of bias was assessed at the study level.

For experimental studies, particularly randomized controlled trials (RCTs), the Cochrane Risk of Bias (RoB) tool was employed, as it is highly recommended for assessing methodological rigor in such studies.

For systematic reviews of observational studies, the Risk of Bias in Non-randomized Studies of Interventions (ROBINS-I) tool was applied. Alternatively, the quality assessment criteria provided by the National Institutes of Health (NIH) were considered, depending on the study design and characteristics.

Only studies classified as having a low risk of bias were included in this review. Studies with a high or unclear risk of bias were excluded to ensure the reliability of the findings.

## 3. Results

**Figure 1:**
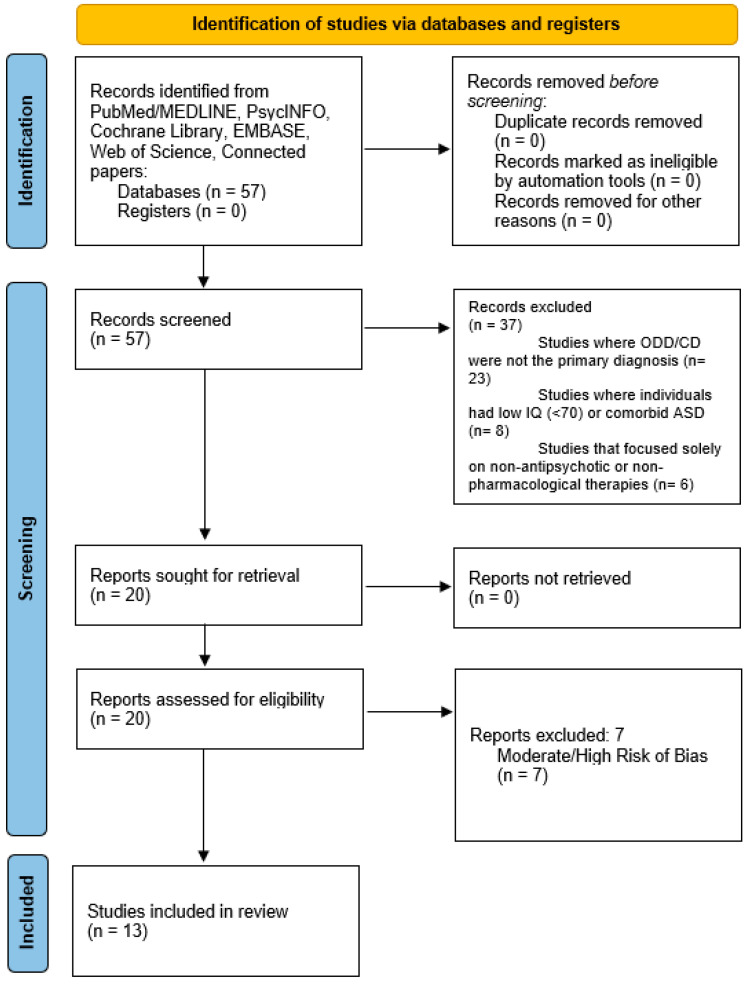
Paper selection flowchart.

Of the 57 studies retrieved for detailed evaluation, 13 were fit for inclusion in the present study. The study selection process is illustrated in Figure 1, following the PRISMA flow diagram.

### 3.1. General Characteristics of the Included Studies

Out of the 13 studies, 4 were RCTs, 2 were longitudinal, 3 were open label, 1 was retrospective, and 3 were systematic reviews with meta-analyses.

The psychopharmacologic therapies investigated predominantly involved atypical antipsychotic medications, particularly risperidone, aripiprazole, and olanzapine. Some trials looked at typical antipsychotics such as haloperidol and chlorpromazine; however, most examined the new atypical agents.

The key characteristics of the included studies—including study type, population, intervention, and outcome measures—are summarized in Table 1.

### 3.2. Narrative Analysis

This systematic review encompassed 13 studies that assessed the efficacy and safety of atypical antipsychotics and various other interventions for managing conduct disorder (CD) and oppositional defiant disorder (ODD) in children and adolescents. A summary of key findings follows.

#### 3.2.1. Atypical Antipsychotics: Efficacy

- Juárez-Treviño et al. (2019) [9]: Clozapine and risperidone were both found to be effective in significantly reducing aggression in children with conduct disorder. Clozapine showed some superior effects on externalizing symptoms and delinquency (*p*-values: 0.039, 0.010, 0.021).
- Pringsheim et al. (2015) [6]: This meta-analysis of 11 RCTs (8 on risperidone, 1 each on quetiapine, haloperidol, and thioridazine) found that atypical antipsychotics significantly reduced disruptive behavior in children with ODD or CD, with an SMD of 0.60 (95% CI 0.31 to 0.89).
- Loy et al. (2017) [10]: This systematic review found atypical antipsychotics to be effective—particularly, risperidone—in reducing irritability and aggression, using tools such as the Aberrant Behavior Checklist to measure outcomes.
- Findling et al. (2006) [15]: Quetiapine produced some significant improvements in aggression and behavioral symptoms among children diagnosed with conduct disorder. Of note was the significant weight gain across subjects that occurred during the trial (4.6 ± 3.9 kg).
- A randomized, double-blind, placebo-controlled study by Connor et al. (2008) [22] found that compared to placebo, quetiapine significantly improved aggressive behaviors, with eight of nine subjects on quetiapine showing clinical improvement (*p* = 0.0006).
- Shafiq et al. (2018) [19]: A systematic review comparing risperidone to placebo in children with conduct problems showed significant reductions in disruptive behavior and aggression, with an SMD of -0.64 (95% CI −0.89 to −0.40). Weight gain was noted in the risperidone group.

#### 3.2.2. Comparative Effectiveness

- Gadow et al. (2016) [11]: A longitudinal study comparing parent training combined with stimulant plus placebo versus stimulant plus risperidone showed both interventions were effective, but the addition of risperidone did not provide significant extra benefit.
- Reyes et al. (2006) [13]: Significantly, the maintenance treatment with risperidone delayed the time to recurrence of symptoms compared to placebo. This underscores the continued treatment imperative for maintained control of aggression and, presumably, for improved psychosocial functioning.
- Findling et al. (2009) [17]: Compared to placebo, aripiprazole reduced aggression scores, but side effects, including sedation, necessitated dose adjustments during the study.
- Masi et al. (2006) [14]: Efficacy was demonstrated with olanzapine in reducing aggression, with 60.9% of patients showing positive response to treatment; however, weight gain was noted.

#### 3.2.3. Augmentation and Combination Therapy

- Gadow et al. (2014) [18]: A 9-week longitudinal study comparing basic stimulant treatment to augmented therapy (stimulant plus risperidone) showed that augmentation provided greater reductions in aggression with peers but had no significant effect on ADHD or ODD symptoms.
- Kronenberger et al. (2007) [16]: After treating the initial stage with methylphenidate, quetiapine was prescribed for the remaining aggressive symptoms in adolescents. This combination resulted in a significant (*p* < 0.01) decrease in aggression scores compared to previous ranks in which only methylphenidate was administered.

#### 3.2.4. Dose-Dependent Efficacy and Long-Term Management

- Findling et al. (2000) [12] carried out a randomized, double-blind, placebo-controlled trial with youths with conduct disorder. They found that risperidone was better than placebo in reducing aggression as measured by the Rating of Aggression Against People and Property (RAAPP).
- Gadow et al. (2014) [18]: A study involving 168 children with severe disruptive behavior reported significant improvements in aggression using parent training combined with stimulant medication. The study underscored the importance of individualized dosing to optimize treatment effects.

#### 3.2.5. Safety and Adverse Effects

- Reyes et al. (2006) [13]: Found that maintenance treatment with risperidone delayed the return of symptoms but was linked to weight gain and other adverse effects. The return of symptoms was significantly delayed in patients who continued risperidone.
- Connor et al. (2008) [22]: Children treated with quetiapine experienced significant weight gain compared to those receiving a placebo. Although it was very effective for managing aggression, quetiapine had some very concerning side effects—profound sedation and an increased appetite.
- Shafiq et al. (2018) [19]: Common adverse effects among children treated with risperidone included weight gain and sedation. However, the authors found risperidone to have significant efficacy in reducing conduct problems in children.

#### 3.2.6. Summary of Effectiveness

In general, atypical antipsychotics, like risperidone, olanzapine, and quetiapine, worked well to reduce aggression and improve behavior in children and adolescents with disruptive behavior disorders. These drugs were effective when used alone (monotherapy) and when combined with other medications (augmentation), especially stimulant medications (e.g., methylphenidate) when children were diagnosed with a disorder like ADHD. Combining these drugs with antipsychotics often led to better outcomes for children and adolescents with serious behavior problems.

#### 3.2.7. Safety Considerations

Adverse events were consistently reported, most notably weight gain, sedation, and metabolic changes, which all potentially impact heart health. Reported weight changes were of special concern, with self-reported weight changes from baseline averaging a gain of about 4 kg across RCTs, this weight gain being highest with olanzapine [14]. Weight gain carries with it a host of negative health consequences and therefore should be taken into account when prescribing these drugs, despite the clear evidence of their efficacy.

## 4. Discussion

### 4.1. Comorbidity and Its Impact on Treatment Outcomes

Due to the complex nature of both CD and ODD, individuals with a primary diagnosis of one of these conditions very commonly have comorbid conditions, namely ADHD, OCD, anxiety and mood disorders [23].

While we tried to minimize the effects of these conditions by only selecting studies where the primary diagnosis was either CD or ODD and excluding those where patients had low IQ or ASD, comorbidities like ADHD complicate the already difficult problem of diagnosing and treating conduct problems for two reasons.

First, they can make the symptoms of opposition and defiance or conduct problems less apparent and thus more difficult to identify.

Second, some studies do not provide enough detail about the management of comorbidities, which limits our understanding of how these conditions might affect the outcomes of the primary treatments being studied [24]. This is an important issue for future research to resolve [25].

### 4.2. Limited-Time Follow-Up

Most studies follow participants for only a few months, which means we have scant data on the effect of these medications on children and adolescents over longer stretches of time. This dearth of data pertains not just to long-term secondary effects, but also to the rate of progression to antisocial personality disorder [26].

### 4.3. Implications for Current Practice

This literature review of existing research on the use of antipsychotic medications in children and adolescents with ODD and CD provides insights into the disorders’ treatment and the medications’ safety, and efficacy. The review includes a wide range of study designs—from RCTs to cohort and observational studies—that together form a picture of the existing evidence.

Moreover, it includes as a primary criterion for inclusion in the analysis that the studies must have ODD or CD as their primary focus. That makes the conclusions drawn in the review more reliable, since it is one of the few reviews that singles out these disorders for treatment with antipsychotics.

Additionally, there is a need for further research into tailored interventions for children and adolescents without comorbidities, as they are often overlooked in existing studies, which tend to focus on more complex cases involving multiple conditions [27].

### 4.4. Stigma in Clinical Practice

One of the surprising things brought to life by this review is the clear stigma that still exists around not only these patients (pediatric population) but also the use of antipsychotic treatment in this age segment [28].

This is clear by the fact that very few studies are conducted on these patients, reflecting a reluctance to study these children and adolescents [10]. The studies that exist are all conducted by a few psychiatrists, while the study of adult populations, especially those with more common and less socially frowned-upon pathologies (like depression) have a much more extensive number of researchers and papers.

As medical professionals, we should strive to minimize our biases and prejudices when conducting scientific research. And even though eliminating them is not possible, we think papers like this one help in shedding light on this reality and in moving the needle [29].

Moreover, this review also shows the hesitation in giving antipsychotic treatment to pediatric populations. This is evidenced by the fact that there already exists plenty of evidence that these medications, when allied with other modalities of treatment, provide the most efficacious treatment of these conditions. So, why are we so unwilling to implement a tool that we have had for so long?

## 5. Conclusions

This systematic review illustrates that pharmacotherapy, especially with clozapine or risperidone, is effective for reducing aggressive and disruptive behavior in children and adolescents diagnosed with ODD and CD. Although clozapine has been studied less frequently, its effectiveness seems to surpass that of risperidone, especially regarding improving delinquency traits and overall functioning. However, this agent also warrants mention in the context of pharmacovigilance because of the potential for serious side effects.

Older “typical” antipsychotics like haloperidol and thioridazine do not seem as effective for these behavioral disorders and have a profile of serious side effects.

Other antipsychotics like quetiapine and ziprasidone, which seem to work well in other populations, remain unstudied and, therefore, their efficacy cannot be determined. Additionally, there is an absence of research on children under the age of five, underscoring a critical gap in the literature.

Overall, antipsychotics are a helpful tool for managing aggression and irritability in children and adolescents with ODD and CD, providing a valuable mechanism of control when other treatment options have failed.

Further large-scale, long-term studies are required to grasp fully the long-term efficacy and safety of these medications.

## Figures and Tables

**Table 1:** Characteristics of the included studies. In each intervention we highlighted the antipsychotic(s) used.

Author and Year	Type of Study	Population	Intervention	Outcome Measurement Tool	Result
Juárez-Treviño et al., 2019 [9]	16-week-long randomized, double-blind, controlled trial	Twenty-four children with conduct disorder aged 6 to 16 years	**Clozapine or Risperidone** Patients were randomized to receive either 0.3 mg/kg of clozapine or 0.025 mg/kg of risperidone on the first visit. Twelve subjects received clozapine and twelve received risperidone. Doses were increased to 0.6 mg/kg for clozapine and 0.05 mg/kg of risperidone during visit two (week one) and were maintained until completion of the trial. The route of administration was by mouth.	The Modified Overt Aggression Scale score was used as the primary outcome of the study. Secondary outcomes were the Child Behavior Checklist (CBCL) externalization (CBCL-E) and internalization factors; Aggression, Hyperactivity, and Delinquency subscales of CBCL-E, Child Global Assessment Scale (CGAS), Barnes Akathisia Rating Scale, and Simpson-Angus Scale.	Both antipsychotics were similarly effective in the primary outcome and in most of the secondary ones. Clozapine was more effective in CBCL-E, the delinquency subscale, and the CGAS scores than risperidone (*p* = 0.039, 0.010, and 0.021).
Loy et al., 2017 [10]	Systematic review with meta-analyses	Ten randomized controlled trials (2000–2014) of atypical antipsychotics versus placebo in children and youths aged up to and including 18 years with diagnoses of disruptive behavior disorders, including comorbid ADHD, involving a total of 896 children and youths	**Risperidone** Atypical antipsychotics versus placebo	Aberrant Behavior Checklist (ABC)–Irritability subscale; the Overt Aggression Scale—Modified (OAS-M) Scale, and the Antisocial Behavior Scale (ABS)	Using the Aberrant Behavior Checklist (ABC)–Irritability subscale, youths treated with risperidone showed reduced aggression compared to youths treated with the placebo (MD −6.49, 95% confidence interval (CI) −8.79 to −4.19; low-quality evidence). When combining the ABS reactive subscale and the OAS-M, the SMD was −1.30 in favor of risperidone (95% CI −2.21 to −0.40, moderate-quality evidence) in comparison to placebo. Of the youths treated with risperidone, only (138 participants) gained 2.37 kilograms (kg) more (95% CI 0.26 to 4.49; moderate-quality evidence) than those on placebo.
Gadow et al., 2016 [11]	52-week-long Longitudinal study	Children with co-occurring attention-deficit/hyperactivity disorder (ADHD), disruptive behavior disorder, and serious physical aggression who participated in a prospective, longitudinal study that began with a controlled, 9-week clinical trial comparing the relative efficacy of parent training + stimulant medication + placebo (Basic; *n* = 84) versus parent training + stimulant + risperidone (Augmented; *n* = 84).	** Risperidone ** Parent training + stimulant medication + placebo versus parent training + stimulant + risperidone	Parent-reported behavioral outcome and Clinical Global Impressions (CGI) severity score	Both randomized groups improved from baseline to follow-up, but the three primary parent-reported behavioral outcomes showed no significant between-group differences. Exploratory analyses indicated Augmented (65%) was more likely (*p* = 0.02) to have a Clinical Global Impressions (CGI) severity score of 1–3 (normal to mildly ill) at follow-up than Basic (42%). Augmented had elevated prolactin levels, and Basic decreased in weight over time.
Findling et al., 2000 [12]	10-week-long randomized, double-blind, controlled trial	Twenty subjects who were outpatients who met DSM-IV criteria for conduct disorder as a primary diagnosis. Other inclusion criteria included being between ages ~5 and 15 years (inclusive), having at least a moderate degree of overall symptom severity as based on the Clinical Global Impression (CGI) Scale.	**Risperidone** Ten youths were randomly assigned to receive placebo, and ten youths were randomly assigned to receive risperidone. Medications could be increased at weekly intervals during the first 6 weeks of the study from an initial dose of 0.25 mg or 0.50 mg each morning, depending on patient wt. Patients weighing less than 50 kg had a maximum total daily dose of risperidone of 1.5 mg. Patients weighing 50 kg or greater had a maximum total daily dose of risperidone of 3.0 mg	Rating of Aggression Against People and Property Scale (RAAPP), Clinical Global Impressions (CGI) severity score	RAAP score difference from baseline: week 10 −0.16 (0.54) −1.65 (0.40) *p* = 0.03; CGI-S difference from baseline: week 10 −0.08 (0.66) −2.58 (0.49) *p* = 0.003
Reyes et al., 2006 [13]	6-month-long randomized, double-blind, controlled trial	Patients with disruptive behavior disorder (5–17 years of age and a range of intellect) who had responded to risperidone treatment over 12 weeks were randomly assigned to 6 months of double-blind treatment with either risperidone or placebo. Treatment was initiated in 527 patients, with 335 randomly assigned to a double-blind maintenance condition.	** Risperidone ** Maintenance versus withdrawal of risperidone treatment	The primary efficacy measure was time to symptom recurrence, defined as sustained deterioration on either the Clinical Global Impression severity rating (/2 points) or the conduct problem subscale of the Nisonger Child Behavior Rating Form (/7 points).	Time to symptom recurrence was significantly longer in patients who continued risperidone treatment than in those switched to placebo. Symptom recurrence in 25% of patients occurred after 119 days with risperidone and 37 days with placebo. Weight increased over the initial 12 weeks of treatment (mean weight z score change = 0.2, SD = 2.7, *N* = 511), after which it plateaued.
Masi et al., 2006 [14]	Retrospective study	Clinical records of the first 23 adolescents diagnosed as having a CD, diagnosed with a clinical interview (K-SADS), either pure or with comorbid diagnoses, and treated with olanzapine	**Olanzapine** All patients were started on a dose of 2.5 mg olanzapine at bedtime, with weekly increase of 2.5–5 mg/day. Drug titration end point (maximum 20 mg/day) was determined by clinical response, age, weight, and side effects. During olanzapine treatment, adjunctive medications doses were held constant.	Modified Overt Aggression Scale (MOAS), Clinical Global Impression-Improvement (CGI-I), and Children Global Assessment Scale (CGAS)	Based on both an improvement of at least 50% at MOAS and a score 1 or 2 at CGI-I, 14 out of 23 patients (60.9%) were classified as responders at the end of the follow-up. Significant improvement at the last observation was found in MOAS (*p* < 0.001) and CGAS (*p* < 0.001) scores. Mean weight gain at the end of the follow-up was 4.6 +/− 3 kg.
Findling et al., 2006 [15]	8-week-long, open-label outpatient trial	Seventeen children of ages 6 to 12 years, with a primary diagnosis of CD	**Quetiapine** Patients weighing less than 35 kg began treatment with a single morning dose of 25 mg quetiapine. Patients weighing more than 35 kg began treatment with 25 mg quetiapine in the morning and 25 mg quetiapine 1 h before bedtime. Each patient’s study medication dose was increased until a total daily dose of approximately 3 mg/kg/day was reached by the end of week 1. This dose was then maintained until the end of week 2. Patients treated with more than 25 mg quetiapine per day were dozed twice daily. If doses were not equally divisible by two, then the higher dose was administered in the morning unless side effects precluded this strategy. After week 2 of PK sampling, patients could have their medication increased to either a total daily dose of 6 mg/kg/day or 750 mg/day (whichever was lower). Dose increases from 25 mg/week to 50 mg/week were permitted up to the end of week 7.	Rating of Aggression Against People and/or Property Scale (RAAPPS), Nisonger Child Behavior Rating Form (NCBRF), and the Conners Parent Rating Scale (CPRS-48).	At the end of week 8, the CPRS T scores showed the following changes compared to baseline: The Conduct score decreased from 92.1 (7.5) at baseline to 70.2 (21.1), with a highly significant improvement (*p* ≤ 0.001). The Learning score dropped from 87.1 (9.3) to 74.5 (16.7), with a highly significant improvement (*p* ≤ 0.001). Psychosomatic scores decreased from 56.0 (13.7) to 51.4 (13.0), although this change was not significant. Impulse scores improved from 75.1 (5.6) to 66.1 (14.3), with a significant improvement (*p* ≤ 0.01). Anxiety scores showed a slight decrease from 50.0 (10.5) to 48.2 (10.4), with no significant difference. Hyperactivity scores dropped from 87.8 (6.7) to 73.8 (17.2), showing a highly significant improvement (*p* ≤ 0.001). For the NCBRF subscales at the end of week 8: The Compliant score increased from 4.7 (2.5) to 5.7 (3.7). Adaptive scores rose from 3.1 (1.6) to 4.6 (2.2). Conduct scores improved significantly, dropping from 37.3 (5.1) to 25.4 (14.4) (*p* ≤ 0.001). Insecure scores decreased from 20.1 (6.3) to 12.8 (8.2), with a highly significant improvement (*p* ≤ 0.001). Hyperactive scores reduced from 20.9 (3.4) to 15.8 (6.4), showing improvement. Self-injury scores decreased from 3.2 (2.6) to 2.5 (3.0). Self-isolated scores improved from 7.5 (4.2) to 5.1 (4.4), with a significant improvement (*p* ≤ 0.01). Sensitive scores decreased from 8.4 (3.4) to 5.7 (3.6), showing a highly significant improvement (*p* ≤ 0.001). Fifteen (88.2%) of the seventeen dosed patients experienced an adverse event during the study. The most frequently reported side effects included the following: fatigue (*n* = 11), nasal congestion (*n* = 8), headache (*n* = 7), nausea (*n* = 4), sedation (*n* = 4), increased appetite (*n* = 4), vomiting (*n* = 3), stomach pain (*n* = 3), irritability (*n* = 2), and fever (*n* = 2). The overall median increase in weight in these 12 patients was 2.3 kg (*p* < 0.001).
Kronenberger et al., 2007 [16]	12-week-long prospective, open-label study	A total of 24 adolescents (aged 12–16 years) with diagnoses of ADHD-combined type and disruptive behavior disorder (n 4 with oppositional defiant disorder; n 20 with conduct disorder)	**Quetiapine** Subjects received 3 weeks of OROS methylphenidate monotherapy titrated to 54 mg/day and then those subjects who remained symptomatic received 9 weeks of adjunctive quetiapine (given b.i.d., with a maximum dose of 600 mg/day).	Rating of Aggression Against People and Property (RAAPP), Modified Overt Aggression Scale (MOAS), MOAS, Clinical Global Impressions–Severity (CGI-S)	For the RAAPP (Rating of Aggression Against People and Property), the baseline score was 4.3 (0.4), and it decreased to 3.2 (0.6) after MPH monotherapy and further to 2.0 (0.8) after the addition of quetiapine. Paired *t*-tests indicated statistically significant reductions, with a t-value of 10.5 (*p* < 0.001) from baseline to MPH and 5.8 (*p* < 0.001) from MPH to MPH + quetiapine. In the MOAS (Modified Overt Aggression Scale Total Score), baseline scores were 229.0 (194.3). After MPH treatment, scores dropped to 73.7 (57.9), and after quetiapine was added, scores decreased further to 26.3 (33.0). Paired *t*-tests showed significant reductions, with a t-value of 4.4 (*p* < 0.001) from baseline to MPH and 3.6 (*p* < 0.01) from MPH to MPH + quetiapine. The CGI-S (Clinical Global Impressions—Severity) score began at 5.3 (0.6) at baseline, decreased to 4.1 (0.8) with MPH, and decreased further to 3.2 (0.9) after quetiapine was added. Paired *t*-tests indicated significant improvements, with a t-value of 6.8 (*p* < 0.001) from baseline to MPH and 6.5 (*p* < 0.001) from MPH to MPH + quetiapine.
Findling et al., 2009 [17]	2-week-long, open label, three-center study	A total of 12 children (6–12 years) and 11 adolescents (13–17 years) with CD and a score of 2–3 on the Rating of Aggression Against People and/or Property (RAAPP)	**Aripiprazole** Initially, the protocol used the following dosing of aripiprazole: subjects < 25 kg, 2 mg = day; subjects 25–50 kg, 5 mg = day; subjects > 50–70 kg, 10 mg = day; and subjects > 70 kg, 15 mg = day. Due to vomiting and sedation, this schedule was revised to <25 kg, 1 mg = day; 25–50 kg, 2 mg = day; >50–70 kg, 5 mg = day; and >70 kg, 10 mg = day.	Rating of Aggression Against People and = or Property (RAAPP), Clinical Global Impressions—Severity (CGI-S) s	On day 1, the mean RAAPP scores were 3.00 ± 0.63 for children and 2.64 ± 0.50 for adolescents. By day 14, both groups had a median RAAPP score of 2, which remained stable through month 36 (children: *p* < 0.05 *p* < 0.05 *p* < 0.05; adolescents: *p* < 0.05 *p* < 0.05 *p* < 0.05). The average decrease in RAAPP scores from baseline to month 36 was 1.00 ± 1.00 for children and 0.75 ± 0.96 for adolescents. The children’s mean CGI-S scores at baseline were 4.27 ± 1.01 (moderately ill), decreasing to 3 (mildly ill) by day 14 (*p* < 0.05 *p* < 0.05 *p* < 0.05) and to 2 (borderline ill) by month 36 (*p* < 0.01 *p* < 0.01 *p* < 0.01). Adolescents showed a similar improvement, with the median CGI-S score decreasing from 4 at baseline to 2 by day 14 (*p* < 0.01 *p* < 0.01 *p* < 0.01) and remaining at this level through month 36. Additionally, by day 14, 63.6% of children and 45.5% of adolescents were rated as “much” or “very much improved” on the CGI-I score, with this percentage increasing to 66.7% of children and 100% of adolescents by month 36 (*p* < 0.01 *p* < 0.01 *p* < 0.01 for both age groups).
Gadow et al., 2014 [18]	9-week-long longitudinal study	A total of 168 children between 6 and 12 years of age with evidence of serious physical aggression, as defined by parent report to a blinded clinician, of a Level 3 or greater Overt Aggression Scale–M (OAS-M) rating of assault against objects (broke several things in anger), others (assault resulting in serious physical injury to another), or self (cut, bruised, burned self but only superficially), and severe disruptive behavior (≥90th percentile NCBRF D-Total); DSM-IV criteria for any subtype of ADHD plus ODD (*n* = 124) or ODD and CD (*n* = 44); and a rating of at least moderately ill by a blinded clinician (severity score ≥ 4 Clinical Global Impression [CGI])	**Risperidone** Open trial of parent training and stimulant medication for 3 weeks. Participants failing to show optimal clinical response were randomly assigned to Basic or Augmented therapy for an additional 6 weeks. At the completion of the baseline assessment, the primary caregiver started parent training, which continued throughout the 9-week intervention, and all children began an open trial of stimulant monotherapy, usually Osmotic Release Oral System (OROS) methylphenidate. If unable to tolerate medication or unable to swallow pills, an alternative stimulant was offered. During the first 3 weeks, the primary clinician adjusted the stimulant to achieve an optimal therapeutic response defined as a CGI-Improvement score of 1 by a blinded clinician and a parent-rated NCBRF D-Total score < 15 (within 0.5 SD of the normative mean). If participants did not show sufficient clinical responses at Week 3, or if they showed deterioration at Week 4 through Week 6 (i.e., dropped below a blinded CGI of 1 or had a NCBRF D-Total > 15), the second agent (risperidone or placebo) was added to the treatment package. The mean Week 9 dose of methylphenidate was 45 ± 15 mg/day (Basic) and 46 ± 17 mg/day (Augmented) (*p* = 0.88). For the second medication, mean doses were 1.9 ± 0.7 mg/day (placebo) and 1.7 + 0.6 mg/day (risperidone) (*p* = 0.07).	Overt Aggression Scale–M (OAS-M), Clinical Global Impression [CGI]	Compared with Basic therapy, children receiving Augmented therapy experienced greater reductions in parent-rated ODD severity (*p* = 0.02, Cohen’s d = 0.27) and peer aggression (*p* = 0.02, Cohen’s d = 0.32) but not ADHD or CD symptoms. Fewer children receiving Augmented (16%) than Basic (40%) therapy were rated by their parents as impaired by ODD symptoms at Week 9/endpoint (*p* = 0.008). Teacher ratings indicated greater reduction in ADHD severity (*p* = 0.02, Cohen’s d = 0.61) with Augmented therapy but not for ODD or CD symptoms or peer aggression.
Pringsheim et al., 2015 [6]	Systematic review with meta-analysis	Eleven RCTs of antipsychotics (eight studied risperidone, one studied quetiapine, one studied haloperidol, and one studied thioridazine); Most studies included youth with ODD or CD, with and without ADHD.	**Risperidone, Quetiapine, Haloperidol or Thioridazine** Antipsychotics vs. placebo	Nisonger Child Behavior Rating Form, the Aberrant Behavior Checklist, the CGI scale, the Child Aggression Scale, the Rating of Aggression Against People or Property Scale, the Conners Parent–Teacher Questionnaire, and the OAS	The SMD between risperidone and placebo for disruptive behavior and aggression was 0.60 (95% CI 0.31 to 0.89; I2 = 0%, *p* < 0.001); CGI-S scores decreased from 5.9 at randomization to 3.4 at end point with quetiapine, compared with a decrease from 5.5 to 5.0 with placebo (*p* = 0.007). The result of Haloperidol was significantly different from placebo on measures of hostility and aggression (magnitude of effect not reported); a small difference between thioridazine and placebo on the Conduct Problems subscale of the Conners Teacher Questionnaire (magnitude of effect not reported).
Shafiq et al., 2018 [19]	Systematic review with meta-analysis	Three RCTs that compare risperidone to placebo for oppositional behavior or conduct problems in children and youth with average IQ	**Risperidone** Risperidone was administered at a dose of 0.5–4.0 mg per day	NCBRF typical IQ Disruptive behavior total, Rating of Aggression Against People and Property (RAAPP), and Conners’ Parent Rating Scale Revised–Long Version (CPRS-L)	The cumulative results of the three trials demonstrated an SMD of −0.64 (95% CI −0.89 to −0.40, I2 = 0%, *p* < 0.00001) (see Figure 1) for the risperidone group compared to the control group after 8–10 weeks of treatment. Jahangard et al. [20] observed a weight gain of 4.2 kg for the risperidone group compared to 0.74 kg for the placebo group. Aman et al. [21] reported a 1.8 kg weight gain in the risperidone group compared to −1.2 kg in the placebo group.
Connor et al., 2008 [22]	7-week, randomized, double-blind, placebo-controlled pilot study	Subjects had to be between the ages of 12 and 17 years inclusive and to meet criteria for a primary psychiatric diagnosis of conduct disorder. In addition, patients had to have a moderate-to-severe degree of aggressive behavior, as documented by an overt aggression scale score ≥ 25 and at least moderate severity of symptoms as documented by a Clinical Global Impressions—Severity (CGI-S) score ≥ 4 at screen. Nine youths were randomly assigned to receive quetiapine, and ten youths were randomly assigned to receive placebo.	**Quetiapine** Quetiapine and placebo were administered on a twice-daily schedule, morning and evening. Dosing began at 25 mg twice daily (b.i.d.) and could be increased by a maximum of 25 mg b.i.d. every 3 days through day 14 of the protocol. Titration was flexible and could be slowed or reduced if adverse events became problematic in the first two study weeks. By day 14, all subjects achieved a daily dose of at least 200 mg quetiapine (100 mg b.i.d.). On day 14, dosing continued to be flexible based on clinician-assessed benefits and patient tolerability. After day 14, dosing could be increased by 50 mg b.i.d. to a daily total of 800 mg (400 mg b.i.d.) at the discretion of the study physician. Dose was titrated until parent report of meaningful clinical benefit or problematic side effects occurred. Total dose was reached at the end of week 5. The dose became fixed for the final 2 weeks of the study.	CGI-S scale and CGI-I scales, OAS rated weekly by parents	At the study end point, eight of the nine subjects randomized to quetiapine were judged improved (CGII ≤ 2), compared to only one of ten subjects randomized to placebo (*p* = 0.0006).

## Data Availability

The data used in the analysis were derived from previously published studies, which can be accessed via the respective sources referenced throughout the manuscript.
